# Transcriptomics and metabolomics reveal the mechanism of metabolites changes in *Cymbidium tortisepalum* var. longibracteatum colour mutation cultivars

**DOI:** 10.1371/journal.pone.0305867

**Published:** 2024-06-25

**Authors:** Yu Jiang, Yaqin Liu, Yang Lin, Xunliang Tu, Junrong He

**Affiliations:** 1 Institute of Horticulture, Sichuan Academy of Agricultural Science, Chengdu, Sichuan, China; 2 Department of Technology Management, Sichuan Academy of Agricultural Science, Chengdu, Sichuan, China; University of Brescia: Universita degli Studi di Brescia, ITALY

## Abstract

**Background:**

Foliage color is considered an important ornamental character of *Cymbidium tortisepalum* (*C*. *tortisepalum*), which significantly improves its horticultural and economic value. However, little is understood on the formation mechanism underlying foliage-color variations.

**Methods:**

In this study, we applied a multi-omics approach based on transcriptomics and metabolomics, to investigate the biomolecule mechanisms of metabolites changes in *C*. *tortisepalum* colour mutation cultivars.

**Results:**

A total of 508 genes were identified as differentially expressed genes (DEGs) between wild and foliage colour mutation *C*. *tortisepalum* cultivars based on transcriptomic data. KEGG enrichment of DEGs showed that genes involved in phenylalanine metabolism, phenylpropanoid biosynthesis, flavonoid biosynthesis and brassinosteroid biosynthesis were most significantly enriched. A total of 420 metabolites were identified in *C*. *tortisepalum* using UPLC-MS/MS-based approach and 115 metabolites differentially produced by the mutation cultivars were identified. KEGG enrichment indicated that the most metabolites differentially produced by the mutation cultivars were involved in glycerophospholipid metabolism, tryptophan metabolism, isoflavonoid biosynthesis, flavone and flavonol biosynthesis. Integrated analysis of the metabolomic and transcriptomic data showed that there were four significant enrichment pathways between the two cultivars, including phenylalanine metabolism, phenylpropanoid biosynthesis, flavone and flavonol biosynthesis and flavonoid biosynthesis.

**Conclusion:**

The results of this study revealed the mechanism of metabolites changes in *C*. *tortisepalum* foliage colour mutation cultivars, which provides a new reference for breeders to improve the foliage color of *C*. *tortisepalum*.

## Introduction

The genus *Cymbidium* is a member of the Orchidaceae, which is one of the largest families of flowering plants [1]. *Cymbidium tortisepalum* (*C*. *tortisepalum*) is a species of the genus *Cymbidium* and an economically important perennial herbaceous plant, which has high ornamental value owing to its foliage, flower color, shape, and fragrance. However, it is threatened by over-collection, habitat disturbance and fragmentation recently [2]. Through classical cross-breeding and mutation breeding, many new cultivars with different traits, such as foliage and flower color, and scent, have been generated [3, 4].

The floral characteristics of *Cymbidium* have previously attracted the most horticultural attention, but leaf variations have recently become of interest. The colorful foliage colors of plants not only attract insect pollination, but also are popular in human society, especially for horticultural plants, such as *C*. *tortisepalum*. There are many factors affecting the foliage color of *Cymbidium*, mainly including genetic factors and environment factor. Previous studies have shown that the synergetic action of multiple factors in vivo and in vitro, such as iron deficiency, flavonoids, carotenoids, and chlorophylls, had influences on the formation of foliage color in different plants including *Cymbidium* [5–7]. For example, iron deficiency would affect the accumulation of physiologically inactive Fe pools in chlorotic leaves through the ferric-chelate reductase activity [8]. Furthermore, flavonoid is also the decisive pigment presented in most foliage colors, among which anthocyanin is the key component. Gao et al. reported that the composition of flavonoids have a close relationship with foliage colors variation in *Cymbidium sinense* ‘Red Sun’ [9].

Compared to other orchids, very little genomic data are available on the regulatory mechanisms of foliage color formation in *C*. *tortisepalum*. Therefore, it was difficult to further study the molecular basis of foliage color. In recent years, transcriptomics and metabolomics have become valuable tools for the characterization of gene expression and elucidating the regulatory mechanisms of foliage color formation at a global level. Transcriptomics could provide detailed information on the gene expression profiling between two or more different samples, whereas metabolomics could generate the metabolites differentially produced by those samples [10, 11]. Therefore, integrated analysis of the transcriptomics and metabolomics could provide valuable and complementary information on the biological processes and mechanisms involved in some characters of samples, which cannot be obtained by using a single omics platform [12].

Therefore, we applied a multi-omics approach based on transcriptomics and metabolomics, to further investigate the biomolecule mechanisms of metabolites changes in *C*. *tortisepalum* colour mutation cultivars. The results obtained by both approaches have led to the identification of a number of functional elements determining foliage color formation and have allowed us to better understand such mechanisms, thus helping orchid breeding and expanding the application field of orchids in the future.

## Materials and methods

### Plant materials and chlorophyll determination

**Fig 1:**
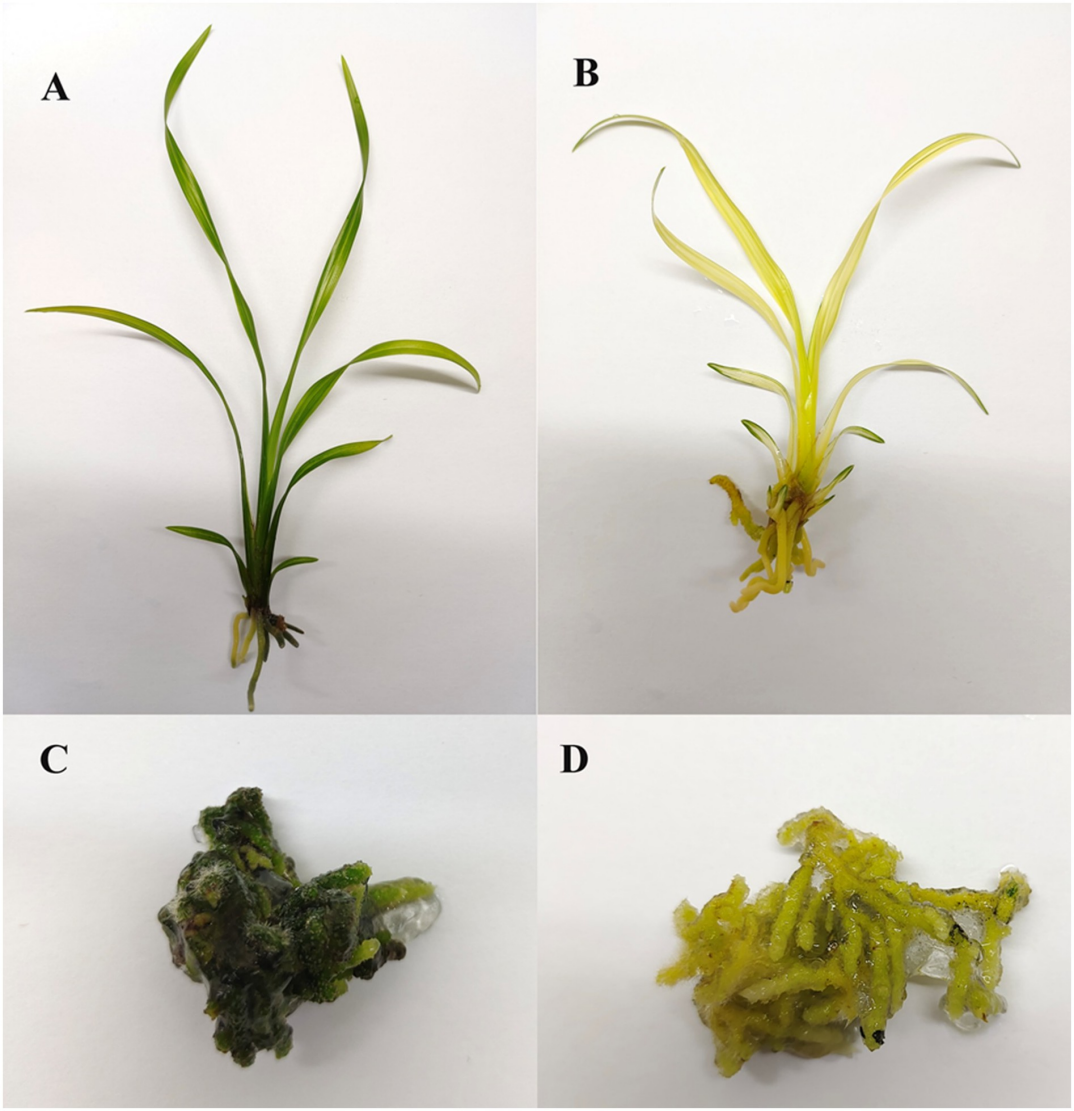
The phenotypes of two *C*. *tortisepalum* cultivars. A and C indicates the seedling and rhizome of wild-type cultivar *C*. *tortisepalum*. B and D indicates the seedling and rhizome of colour mutation cultivar *C*. *tortisepalum*.

The wild-type cultivar *C*. *tortisepalum* var. longibracteatum “Longchangsu” (Fig 1A and 1C) and its colour mutation obtained from 60Co γ radiation inducing (Fig 1B and 1D) were used in the present study [13]. The rhizomes of two cultivars (the green rhizome was named GR and the yellow rhizome was named YR) were inoculated into MS culture medium (pH = 6.0) for 180 days with controlled temperatures as previously described in Jiang et al (2022). Four independent biological replicates and three independent biological replicates were processed for transcriptomics and metabolomics analysis. The samples used for metabolomics were the same with transcriptomics and immediately frozen in liquid nitrogen. The UV-VIS spectrophotometry method was used to determinate the content of chlorophyll according to the description of Peng et al [14]. No ethical approval is required.

### RNA extraction, library construction and RNA-sequencing

After collecting rhizomes from two cultivars, the total RNA was extracted using Plant RNA Purification Reagent (Invitrogen, USA) following the manufacturer’s instructions and treated with DNase I (Fermentas, Canada). The concentration and purity of the total RNA were assessed using a Nanodrop 2000, and the RNA integrity was evaluated by 1.5% agarose gel electrophoresis. The 12 independent cDNA libraries mentioned above for transcriptomics were constructed as described in previous studies [15]. The mRNA was isolated and purified according to the manufacturer’s protocol. Then, the mRNA was fragmented into about 200 bp pieces using metal ion. Reverse transcriptase and N6 random hexamers were used to synthesize the first-strand cDNA. Using dNTPs, DNA Polymerase I and RNase H, second strand cDNA synthesis was performed. The products were ligated to Illumina sequencing adapters and further enriched with PCR for preparing the final sequencing library [16]. The cDNA library was sequenced using the Illumina HiSeq 4000 platform (Illumina, USA) at the Shanghai Majorbio Bio-pharm Technology Co., Ltd. (Shanghai, China).

### De novo assembly and gene annotation

Raw reads with adaptors, low-quality bases and reads containing more than 10% of unknown nucleotides (N) were first filtered to get clean reads. De novo assembly of clean reads was performed using the Trinity program to obtain unigenes [17]. Potential coding regions were analyzed using the protocol of Trinity software. Length distribution was analyzed using common perl scripts. The N50 length, N90 length, average length and unigene numbers with different length intervals were all calculated.

The functions of the unigenes were annotated using BlastX (V 2.10.0+) with a threshold of E-value of 1e-5 against protein databases, including the non-redundant database, Swissprot protein database and KEGG database [18]. The proteins with the highest sequence similarity were retrieved for analysis.

### Abundance estimation and differential expression analysis

To investigate the expression profile of unigenes between samples, the perl scripts in Trinity package were used to normalize the expression levels for each unigene to obtain fragments per kilo base per million (FPKM) values. The final expression level of unigenes in each treatment was defined as the average value of four independent biological replicates.

Differential expression analysis between samples was performed using edgeR to identify differentially expressed genes (DEGs) [19]. DEGs between samples were considered to be significantly different with an FDR (false discovery rate) <0.05, and an absolute value of log2fold-change (log2FC) ≥1, according to the method described by Lv et al. [20].

### qRT-PCR validation of DEGs

To validate the expression level results from RNA-Seq analysis, five DEGs involved in flavonoid biosynthesis were performed qRT-PCR validation. The template cDNA using for qRT-PCR was obtained from materials in transcriptomics. The detailed method of qRT-PCR refers to Lv et al (2021b). Primers used in this study designed by Primer Premier 5 and listed in S1 Table. The comparative 2^−ΔΔCT^ method was employed to calculate relative expression levels between the target genes.

### Sample preparation and metabolite extraction

The freeze-dried GR and YR samples were crushed using a mixer mill (MM 400, Retsch) with zirconia beads for 1.5 min at 30 Hz. Metabolites were extracted as previously described [21]. 100 mg samples were powdered and extracted overnight at 4°C with 600 ul 70% aqueous methanol. Then, samples were collected by centrifugation at 12,000 rpm and 4°C for 10 min, and the extracts were absorbed and filtrated before UPLC-MS/MS analysis.

### UPLC-MS/MS analysis

Metabolite extractions were analyzed using an UPLC-ESI-MS/MS system. Waters ACQUITY UPLC HSS T3 C18 (1.8 μm, 2.1 mm*100 mm) column was used for UPLC. Water, acetonitrile, and pure water with 0.04% acetic acid were set as mobile phase. A gradient program that employed the starting conditions of 95% water and 5% acetonitrile was used for sample measurements. The column oven was set to 40°C and the injection volume was 4 μl.

Linear ion trap (LIT) and triple quadrupole scans were acquired on a triple quadrupole-linear ion trap mass spectrometer (Q TRAP), API 4500 Q TRAP UPLC/MS/MS System. Instrument tuning and mass calibration in triple quadrupole and LIT modes used 10 and 100 μmol/L polypropylene glycol solutions, respectively. Triple quadrupole scans were acquired as multiple reaction monitoring (MRM) experiments [22].

### Quantitative analysis and KEGG annotation of metabolites differentially produced by the mutation cultivars

Metabolites differentially produced by the mutation cultivars were determined by variable-importance-projection (VIP) ≥1 and absolute log2FC (fold change) ≥1. VIP values were extracted from orthogonal partial least squares-discriminant analysis (OPLS-DA) result, which was generated by R package MetaboAnalystR [23]. The data was log transform (log2) and mean centering before OPLS-DA. Identified metabolites were annotated using KEGG Compound database (http://www.kegg.jp/kegg/compound/), and annotated metabolites were then mapped to KEGG pathway database (http://www.kegg.jp/kegg/pathway.html) [24].

## Results

### The growth and chlorophyll content of two *C*. *tortisepalum* cultivars

The two *C*. *tortisepalum* cultivars showed a significant phenotype differences. As shown in Fig 1, the rhizomes and leaves of color mutation is bright yellow, while the wild-type shows a dark green colour. In addition, the average multiplication weight and seedlings height of color mutation were 8.15 g and 5.68 cm, while these of wild-type were 11.47 g and 9.07 cm. Furthermore, the content of chlorophyll was determined and the results showed that the content of chlorophyll was significantly decreased in the color mutation cultivars (Table 1).

**Table 1:** The growth and chlorophyll content of two *C*. *tortisepalum* cultivars.

	Wild-type	Colour mutation
Average multiplication weight (g)	11.47	8.15
Seedlings height (cm)	9.07	5.68
Chlorophyll a (mg/g)	1.383±0.007	0.049±0.010
Chlorophyll b (mg/g)	0.550±0.011	0.023±0.002
Chlorophyll a+b (mg/g)	1.935	0.073

### Sequencing and de novo assembly of *C*. *tortisepalum* transcriptome

We performed a transcriptome analysis of two pools (wild-type: GR and color mutation: YR) rhizomes of mRNA samples, generating a total of 9.23 GB raw reads with a Q30 percentage (sequencing error rate 0.1%) above 90.11%. After filtering, 3.76 GB and 4.82 GB clean reads were obtained from GR and YR libraries. The filtered reads were de novo assembled and the assembly results revealed that the transcriptome of *C*. *tortisepalum* consists of 116406 unigenes. The mean length of these unigenes was 598 bp and the N50 value was 896 bp. The size distribution of the unigenes was shown in S1 Fig. These results showed that the quality of the assembled transcriptome is good enough for further analysis. The high-quality reads have been deposited in the NCBI SRA database (accession number: PRJNA471405).

### Identification and functional annotation of DEGs

**Fig 2:**
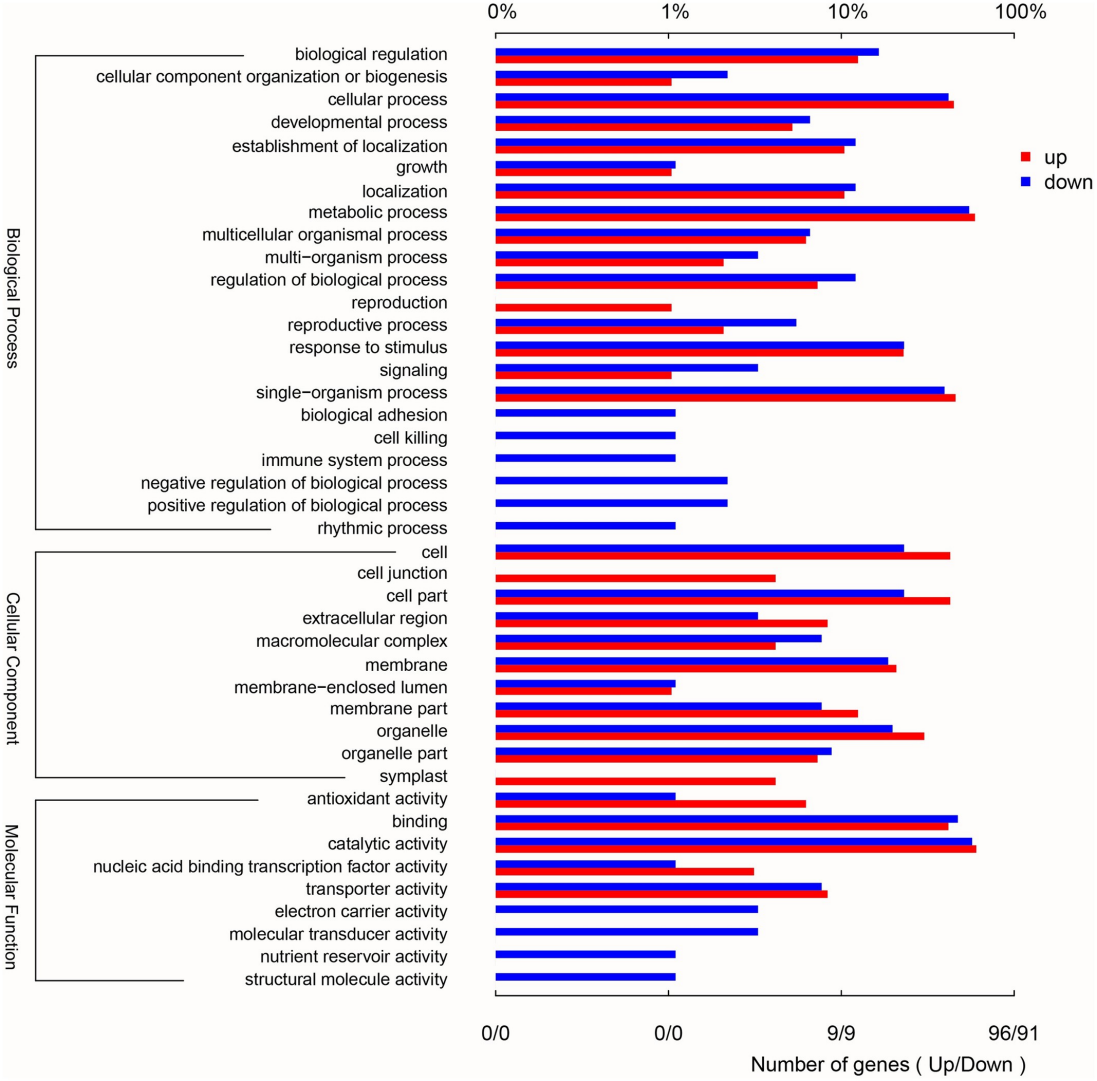
Gene Ontology classification and enrichment of the DEGs. The y-axis represents the GO term and enrichment, and the x-axis represents the percentage of genes (top) and the number of DEGs (bottom). GO annotation of DEGs was grouped into three major categories: biological processes, cellular components and molecular functions.

On the basis of the applied criteria [FDR <0.05, (log2FC) ≥1], 508 genes were identified as DEGs between GR and YR, in which 224 were up-regulated genes and 284 were down-regulated genes. For these DEGs, GO and KEGG analysis were performed. GO annotation of DEGs was grouped into three major categories: biological processes, cellular components and molecular functions. Terms of ‘reproduction’, ‘cell junction’ and ‘symplast’ were only enriched in up-regulated genes, while ‘biological adhesion’, ‘rhythmic process’, ‘electron carrier activity’, ‘nutrient reservoir activity’ and so on were only enriched in down-regulated genes (Fig 2).

**Fig 3:**
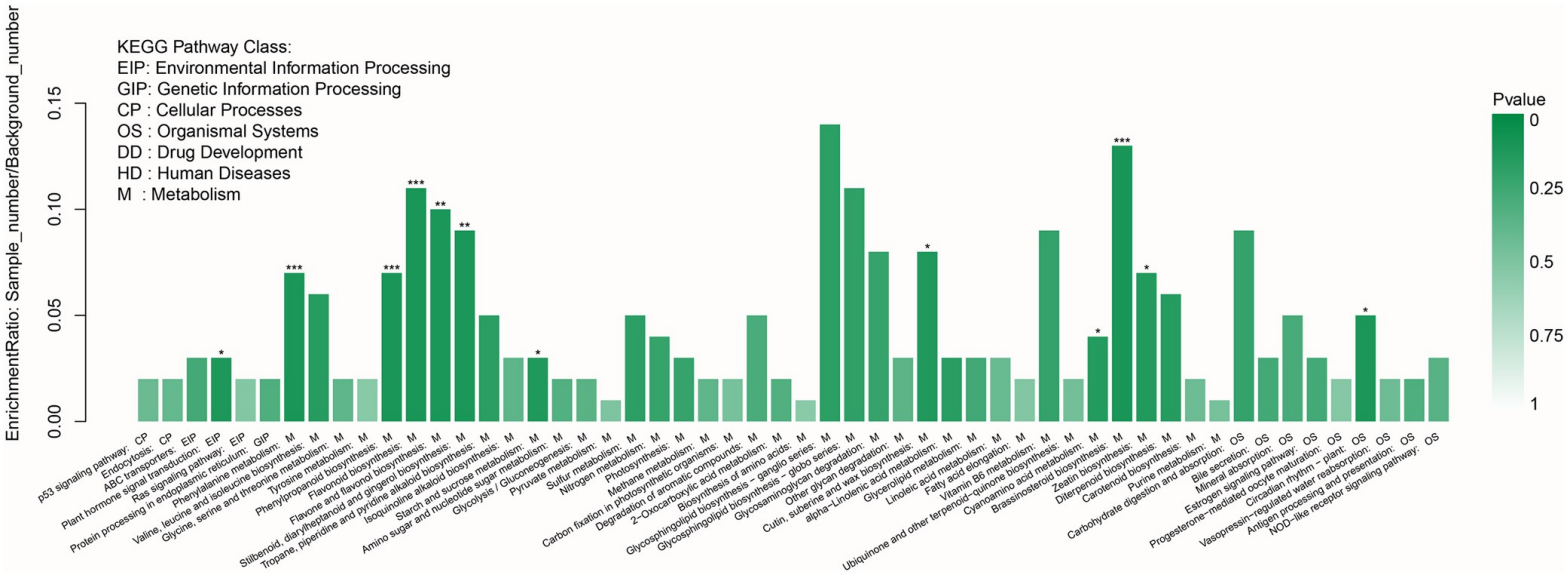
KEGG enrichment of the DEGs. The y-axis represents the enrichment ratio, and the x-axis represents the KEGG pathway class.

To further investigate the biochemical pathways of these DEGs, we mapped all DEGs to the KEGG database. Fig 3 shows the top 80 enriched KEGG pathways. It is obviously observed that most of the top enriched KEGG pathways were classified into metabolism. Of those, four pathways were most significantly enriched (corrected P value ≤0.001): phenylalanine metabolism, phenylpropanoid biosynthesis, flavonoid biosynthesis and brassinosteroid biosynthesis, which suggested that metabolisms may be significantly different between the two cultivars.

### Qualitative and quantitative analysis of metabolites

**Fig 4:**
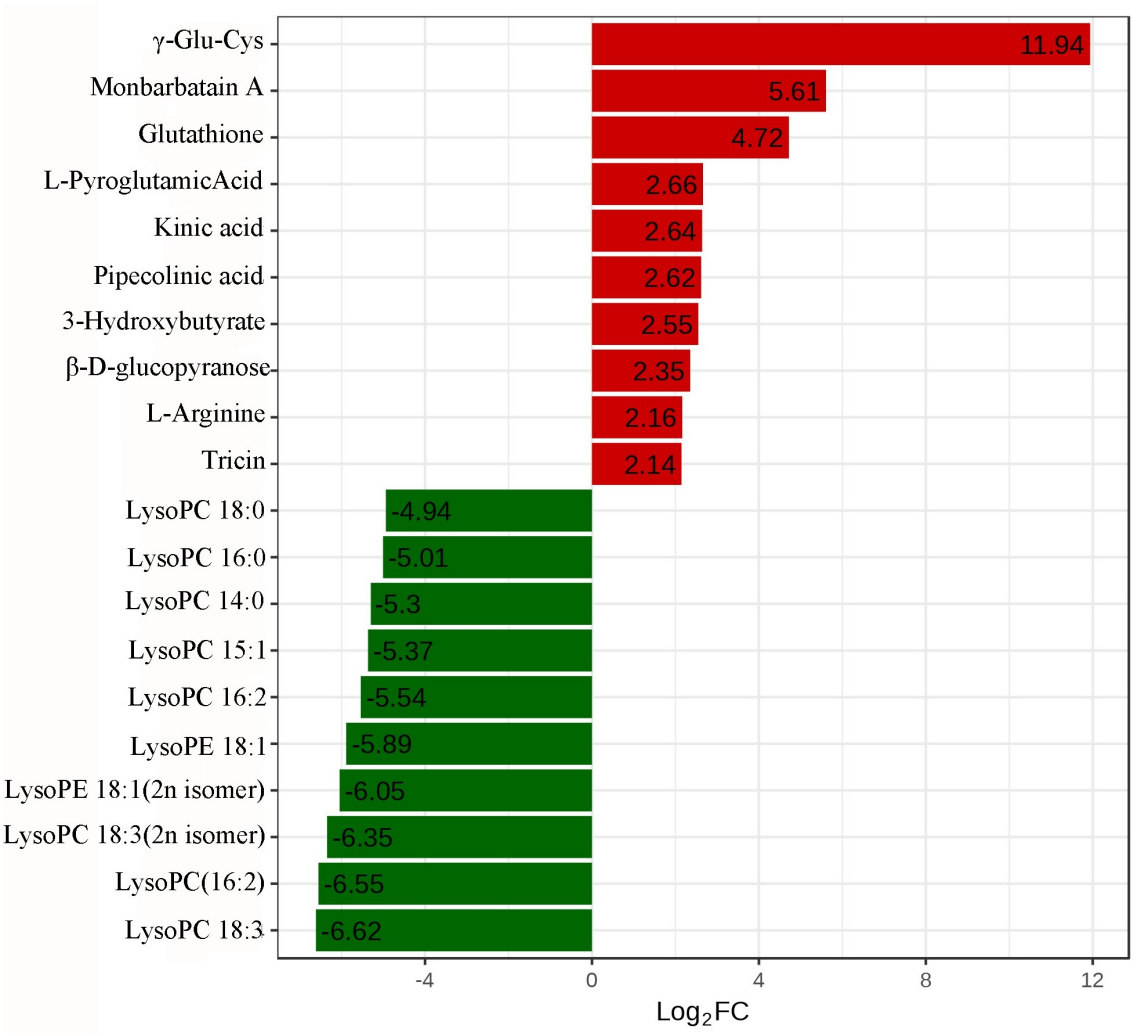
The top 10 up-regulated and down-regulated metabolites. LysoPC indicates lysophosphatidyl choline; LysoPE indicates lysophosphatidyl ethanolamine.

To investigate the metabolites differentially produced by the two *C*. *tortisepalum* cultivars, UPLC-ESI-MS/MS system were used. Through this method, a total of 420 metabolites were identified in *C*. *tortisepalum*. Before analyzing the metabolites differentially produced by the two *C*. *tortisepalum* cultivars, principal component analysis (PCA) was performed and the results showed a preferable clustering among the three biological replicates (S2 Fig). The results of OPLS-DA further confirmed the preferable clustering (S3 Fig). On the basis of the applied criteria (p < 0.05), 115 metabolites differentially produced by the two cultivars were identified, including 37 up-regulated and 78 down-regulated metabolites. Fig 4 shows the top 10 up-regulated and down-regulated metabolites. From the results, the up-regulated metabolites are mainly amino acid and its derivatives, organic acid and flavonoid, while the down-regulated metabolites were mainly lipids.

### KEGG enrichment of metabolites differentially produced by the mutation cultivars

**Fig 5:**
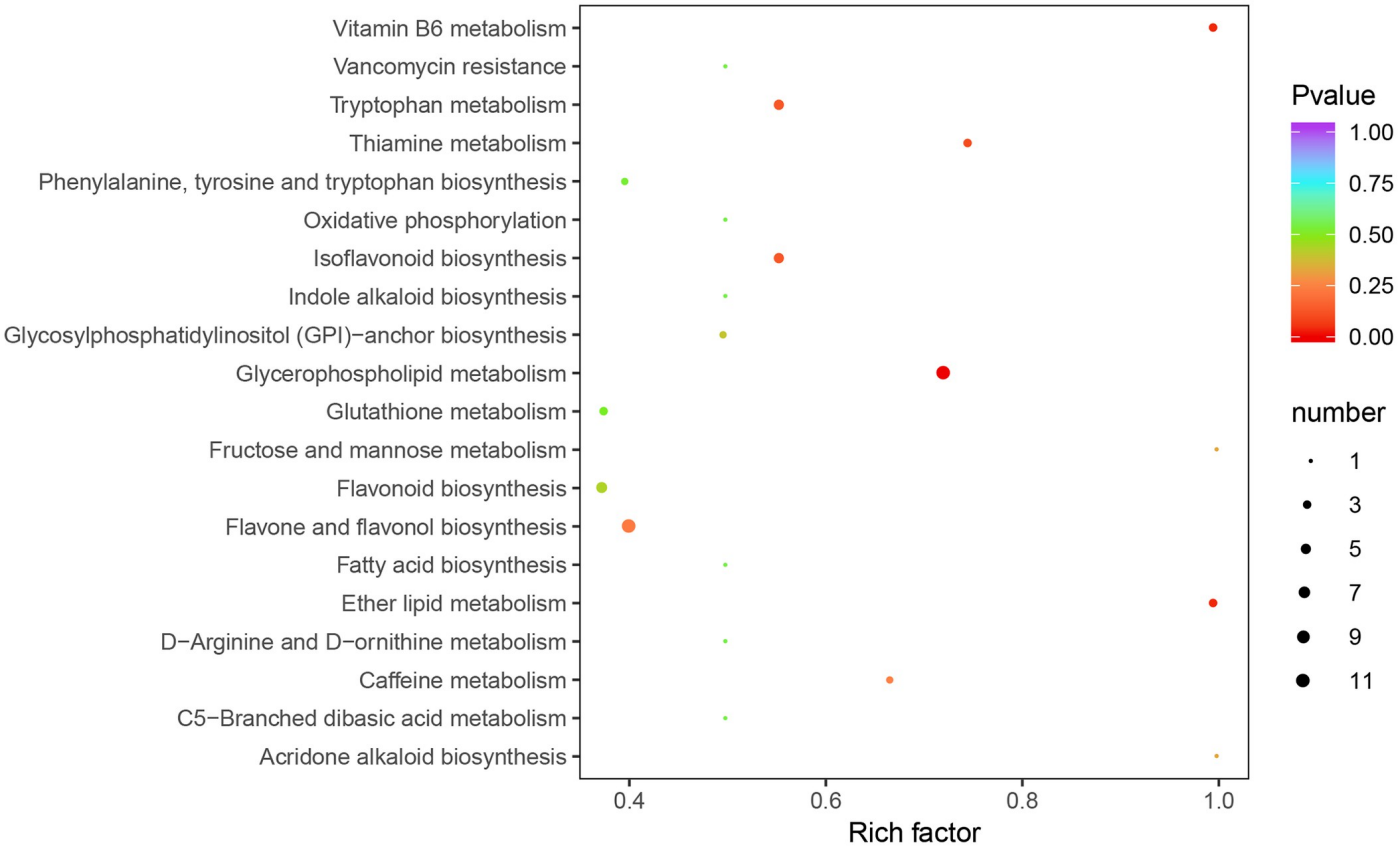
The functional enrichment analysis of differential metabolites using KEGG annotation. The x-axis represents the rich factor, and the y-axis represents the pathway name.

To further investigate the biochemical pathways of these metabolites differentially produced by the mutation cultivars, all differential metabolites were mapped to the KEGG database. KEGG enrichment indicated that the most metabolites differentially produced by the mutation cultivars significantly enriched in the pathway of glycerophospholipid metabolism, tryptophan metabolism, isoflavonoid biosynthesis, flavone and flavonol biosynthesis and ether lipid metabolism (Fig 5). The results are in accordance with the qualitative and quantitative analysis of metabolites, in which most of metabolites differentially produced by the mutation cultivars were involved in amino acid and its derivatives, lipids and flavonoid.

### Integrated analysis of the transcriptomics and metabolomics

**Fig 6:**
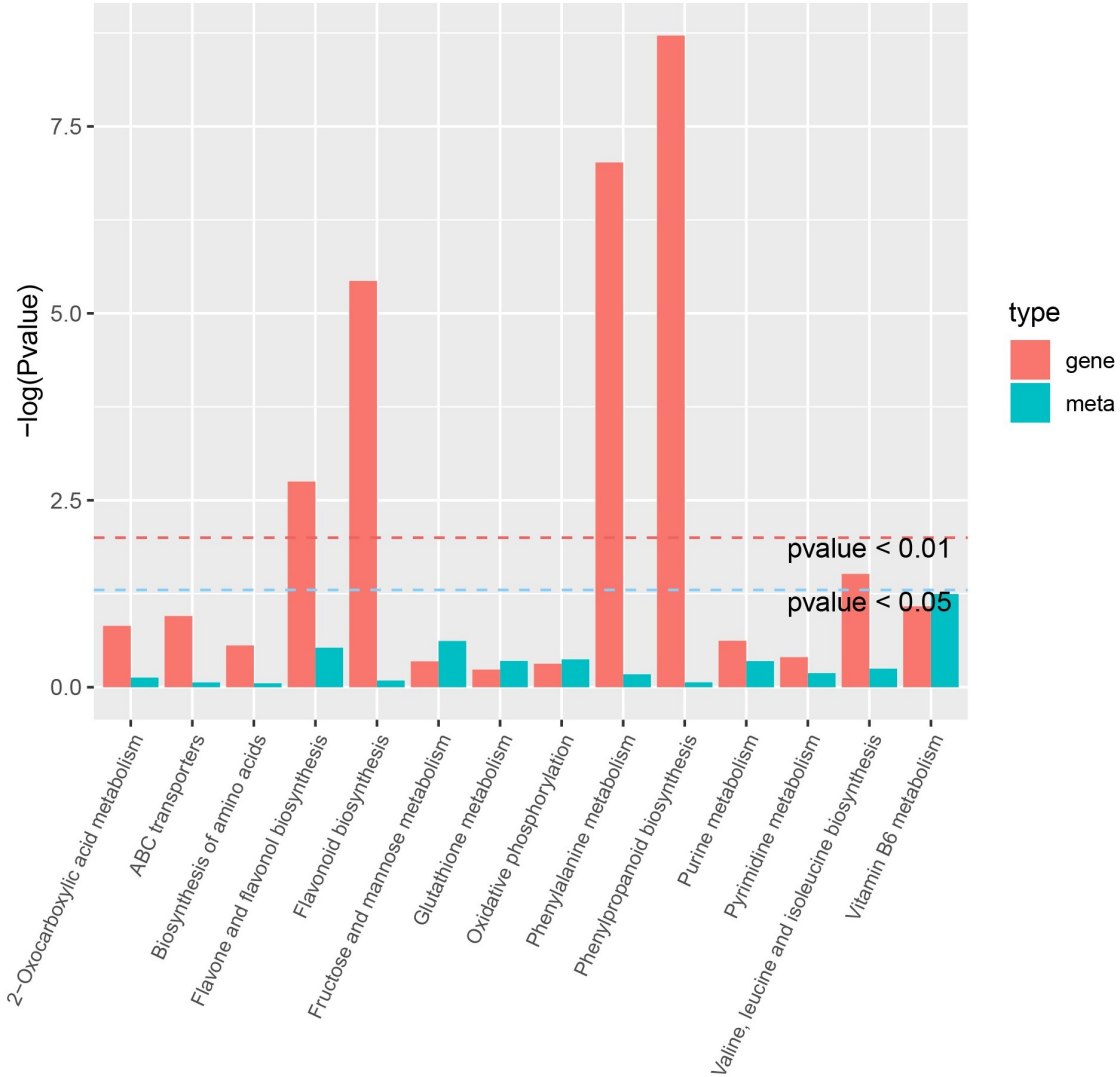
The top 14 enriched KEGG pathways of metabolite-gene pairs between GR and YR. The red bars indicates genes and the blue bars indicates metabolites.

To further investigate the difference between GR and YR, integrated analysis of the metabolomic and transcriptomic data was performed. Top 14 enriched KEGG pathways of metabolite-gene pairs between GR and YR are shown in Fig 6. The result showed that there were four significant enrichment pathways between GR and YR (p < 0.01), including phenylalanine metabolism, phenylpropanoid biosynthesis, flavone and flavonol biosynthesis and flavonoid biosynthesis. In addition, metabolite-gene pairs involved in valine, leucine and isoleucine biosynthesis were enriched.

### Differential genes and metabolites involved in flavonoid biosynthesis

**Fig 7:**
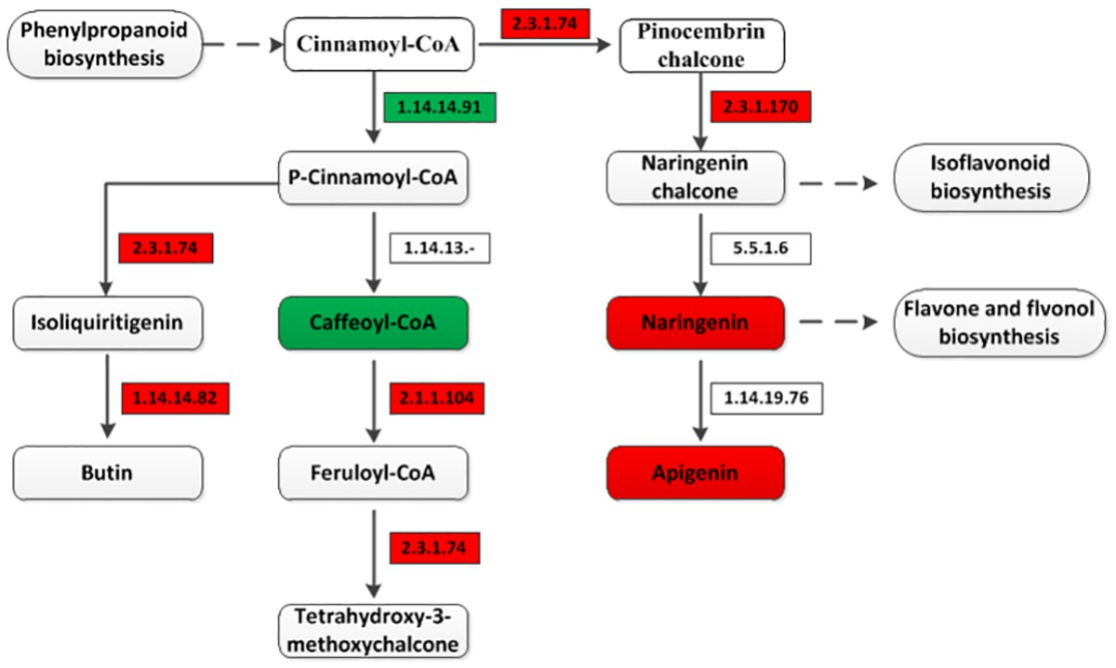
Differential genes and metabolites involved in flavonoid biosynthesis.

Flavonoids are a major class of plant secondary metabolites that serves a multitude of functions including pigments and antioxidant activity. Therefore, differential genes and metabolites involved in flavonoid biosynthesis were identified in this study according to the KEGG pathway. A total of five differential genes and three metabolites were identified in this pathway (Fig 7). For these five DEGs involved in flavonoid biosynthesis, qRT-PCR validation were performed and the results showed a consistency with RNA-seq. Flavonoids are synthesized from cinnamoyl-CoA which is derived from phenylpropanoid biosynthesis. Then, cinnamoyl-CoA results in naringenin chalcone with a diphenylpropane unit, which is converted to naringenin with the flavone backbone by conjugate ring closure. These and further modifications yield a variety of structural forms including apigenin, flavones and flavonols, and isoflavonoids. At the same time, cinnamoyl-CoA results in butin under chalcone synthase (2.3.1.74) and flavonoid 3’-monooxygenase (1.14.14.82) and tetrahydroxy-3-methoxychalcone under chalcone synthase (2.3.1.74) and caffeoyl-CoA O-methyltransferase (2.1.1.104) as well. In this pathway, caffeoyl-CoA was down-regulated while naringenin and apigenin were up-regulated.

## Discussion

*C*. *tortisepalum* is an economically important perennial herbaceous plant due to its colourful flowers and its colourful, elegant, upright foliage. Most of researchers focus on the floral characteristics of *Cymbidium* previously, but leaf variations have recently become of interest. Foliage color variation could improve the horticultural and economic value of *Cymbidium* and has become one of the main focuses of its cultivation and breeding [25]. However, little is known about the control of foliage color of *Cymbidium*. It is well known that flavonoids are the main metabolites affecting foliage color in many plants. Flavonoids were widely accumulated in plant cell vacuoles and distributed throughout the plant kingdom. For example, Nguyen and Cin found that *Solenostemon scutellarioides* (L). Codd varieties change pigmentation in foliage when exposed to high light intensity resulting from the flavonoid changes [26]. The color of camphor tree red bark mutant ‘Gantong 1’ changed accompanying with the changing of flavonoid content by integrated analysis of transcriptomics and metabolomics. Furthermore, with the spraying of salicylic acid and sucrose, a significant correlation of foliage color and flavonoid biosynthesis was observed in *Pistacia chinensis* leaves [27]. In our study, integrated analysis of the transcriptomics and metabolomics showed that pathways involved in flavone and flavonol biosynthesis and flavonoid biosynthesis were significant enriched between wild-type and color mutation *C*. *tortisepalum*. These results confirmed that flavonoids affect foliage coloration in *C*. *tortisepalum* as well. In addition, it is observed from integrated analysis of the transcriptomics and metabolomics that pathways involved in phenylalanine metabolism and phenylpropanoid biosynthesis were significant enriched as well. We inferred that phenylalanine metabolism and phenylpropanoid biosynthesis may affect foliage coloration via flavonoid biosynthesis. As an aromatic amino acid, phenylalanine was generated through the shikimic acid pathway and provides the essential 3-carbon side chain and 6-carbon ring for all phenylpropanoids. Furthermore, phenylalanine, the precursor of cinnamic acid, eventually produce most of flavonoids in plants [28]. After the treatment of phenylalanine for 40 days, the content of flavonoids was increased in *Boesenbergia rotunda* cell suspension culture [29]. In different color of ornamental crabapple, the activities of phenylalanine ammonia lyase, cinnamic acid-4-hydroxylase and 4-coumaryl CoA ligase, which were the main enzymes in transforming phenylalanine into cinnamic acid, were differentially expressed [30].

In addition, *Cymbidium* has important potential medicinal value in the traditional system of medicine. For example, aerial roots were reported to be used for joining fractured bones and whole plant were reported to be used as emetic, tonic and in treating ear-ache, burns and sores [31, 32]. Furthermore, flavonoids were not only affect foliage coloration but also have a good effect on clinical application, including anti-oxidant activity and Parkinson’s disease, even SARS-CoV-2 [33, 34]. Additionally, phenylpropanoids also have many beneficial functions for human health including anticancer and anti-inflammatory properties [35]. In the current study, pathways involved in flavonoid biosynthesis and phenylpropanoid biosynthesis were significant enriched between GR and YR, which suggested that the colour mutation of *C*. *tortisepalum* was not only highlight the value of ornamental but also increase its economic value or potential medicinal value. It is well known that genetic engineering techniques were the efficient method to enhance the value of plants. For example, Makhtoum et al. are focused on QTLs on important characteristics related to abiotic tolerance in barley [36]. Yousefi et al. characterized a new ecotype of holoparasitic plant *Orobanche* L. on host weed *Xanthium spinosum* L [37]. There researches and this study provide a new reference for breeders to improve the foliage color of *C*. *tortisepalum*.

## Conclusion

This study investigated the biomolecule mechanisms of metabolites changes in *C*. *tortisepalum* colour mutation cultivars. The ability to proliferate and differentiate in mutation cultivars was decreased and the contents of chlorophyll and carotenoids were significantly decreased as well. However, the value of carotenoids/chlorophyll in mutation cultivars was higher than that of control group. The integrated analysis of the transcriptomics and metabolomics showed that the biomolecule mechanisms of metabolites changes in *C*. *tortisepalum* colour mutation cultivars may be resulted by gene expression and metabolites changes in phenylalanine metabolism, phenylpropanoid biosynthesis, flavone and flavonol biosynthesis and flavonoid biosynthesis. Our study provides a new reference for breeders to improve the foliage color of *C*. *tortisepalum*.

## Supporting information

S1 Fig Length distributions of *C*. *tortisepalum* transcriptome. The y-axis represents the number of unigenes in the length range, and the x-axis represents the length range of unigenes.
(TIF)

S2 Fig Principal component analysis of metabolites. (TIF)

S3 Fig Orthogonal partial least squares-discriminant analysis of metabolites. (TIF)

S1 Table qRT-PCR Primers used in this study. (DOCX)
