# Supplementary figures and images for: Transcriptomics and metabolomics reveal the mechanism of metabolites changes in Cymbidium tortisepalum var. longibracteatum colour mutation cultivars
Source: PLoS One. 2024 Jun 25;19(6):e0305867. doi: 10.1371/journal.pone.0305867 (PMC11198847; doi:10.1371/journal.pone.0305867)

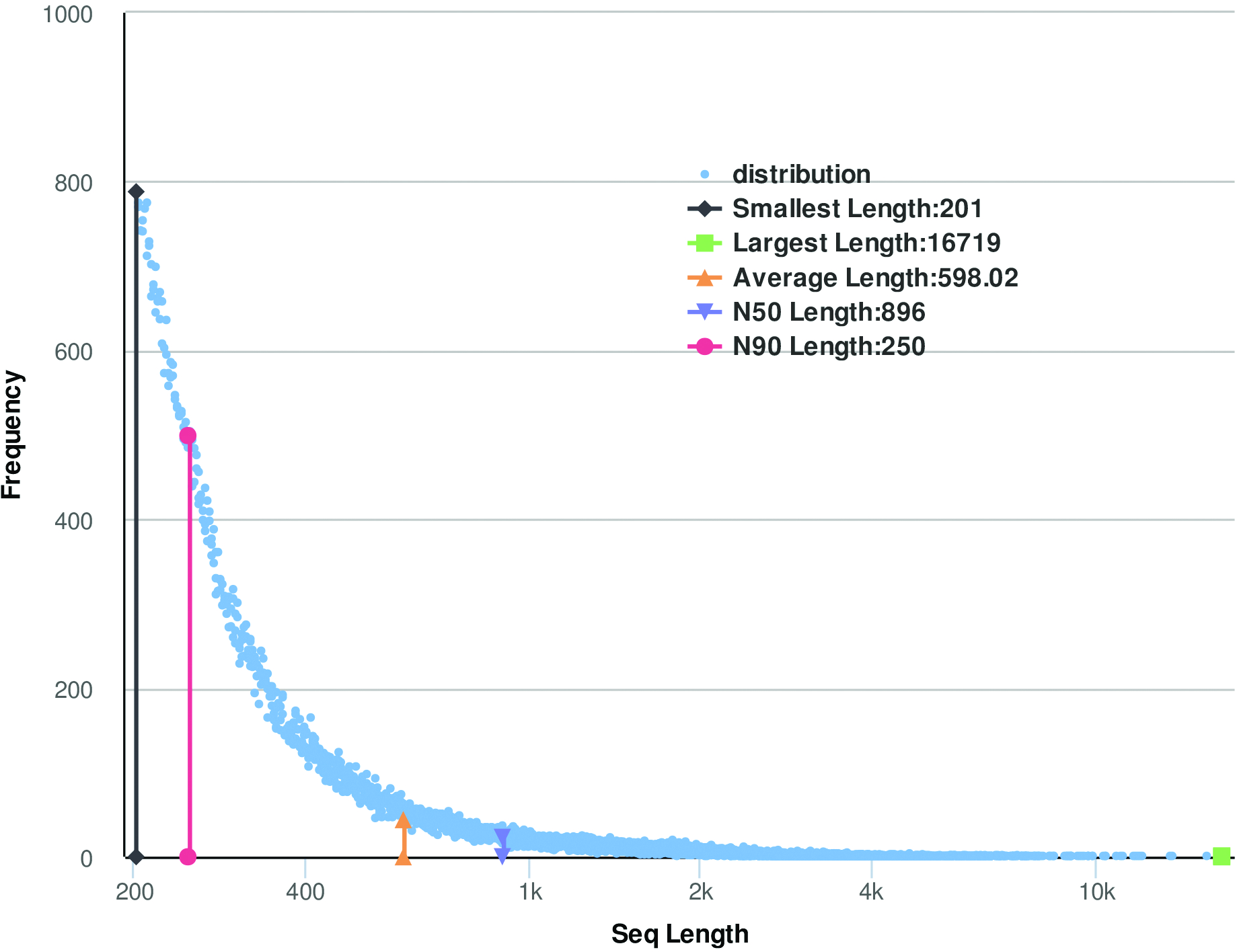

Supplement: S1 Fig — The y-axis represents the number of unigenes in the length range, and the x-axis represents the length range of unigenes. (TIF) [file pone.0305867.s001.tif]

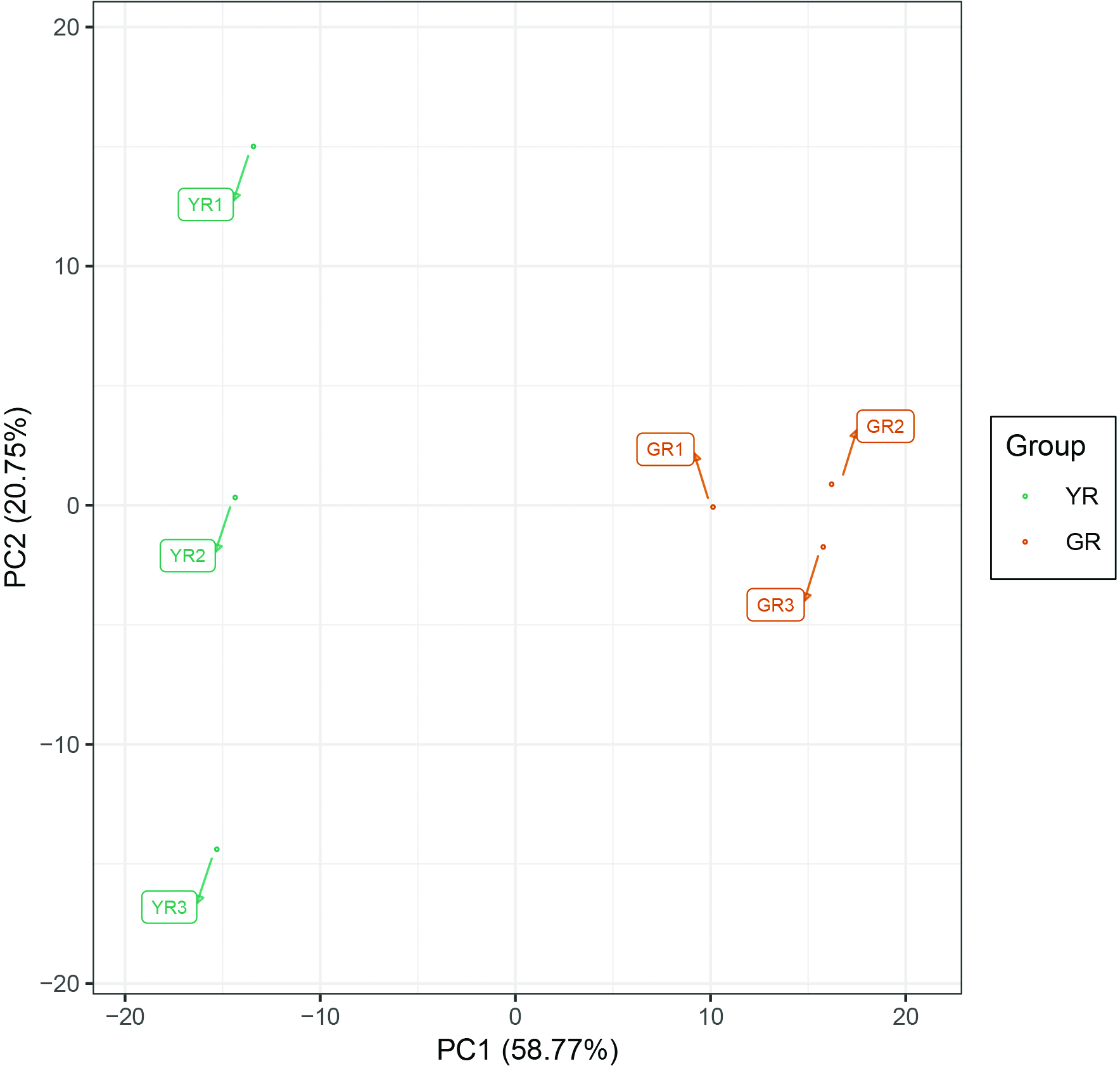

Supplement: S2 Fig — (TIF) [file pone.0305867.s002.tif]

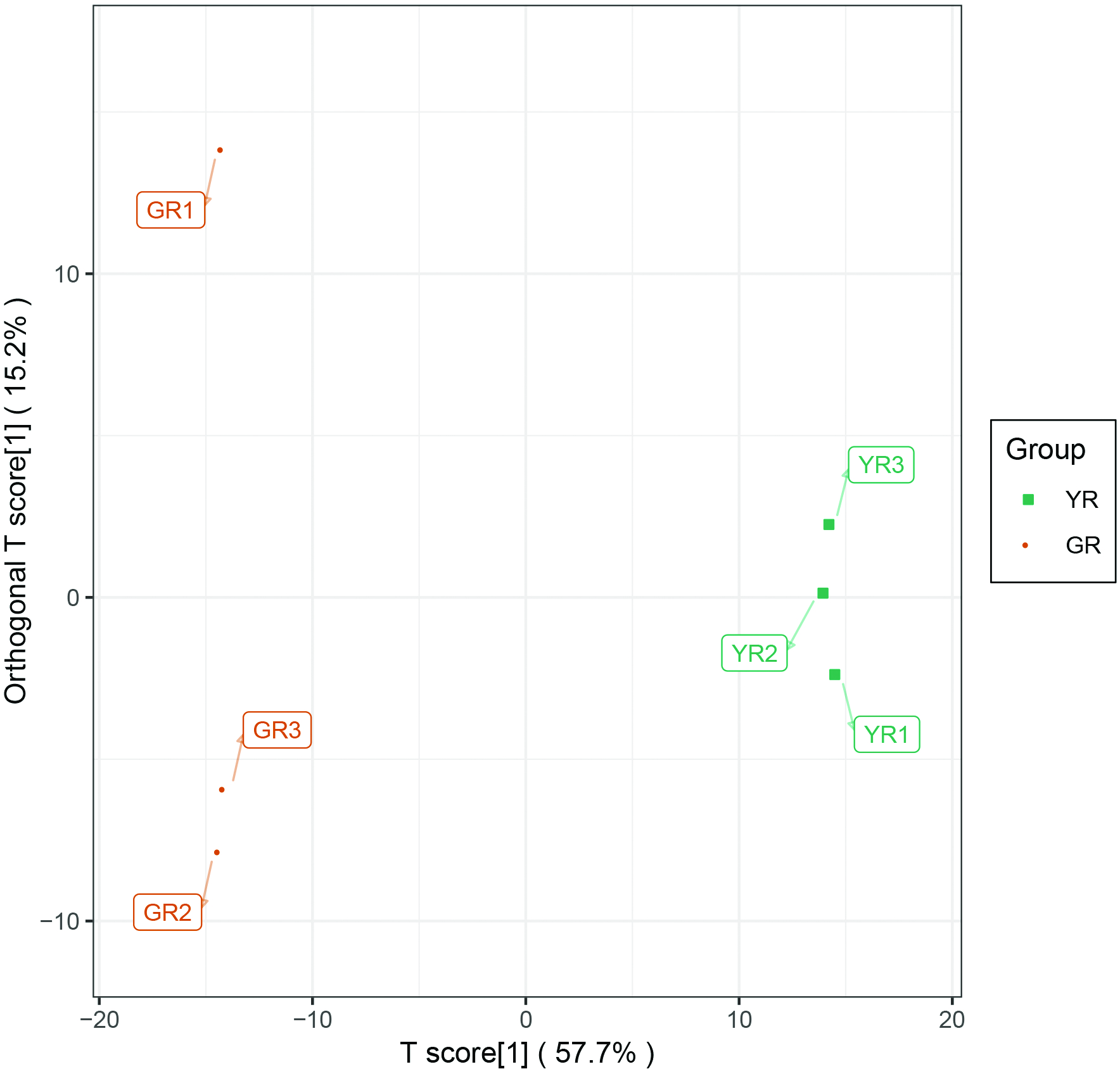

Supplement: S3 Fig — (TIF) [file pone.0305867.s003.tif]
