# Supplementary material for: Transcriptomics and metabolomics reveal the mechanism of metabolites changes in Cymbidium tortisepalum var. longibracteatum colour mutation cultivars
Source: PLoS One. 2024 Jun 25;19(6):e0305867. doi: 10.1371/journal.pone.0305867 (PMC11198847; doi:10.1371/journal.pone.0305867)
Supplement: S1 Table — (DOCX) [file pone.0305867.s004.docx]

**Table S1. qRT-PCR Primers used in this study**

| **Primer** | **Sequence (5′-3′)** | **Purpose** |
| --- | --- | --- |
| c22242_g1-F | CGAGATCATTGCCGAGATGAA | qRT-PCR of 1.14.14.82 |
| c22242_g1-R | GTGTAGTCGTGGTTGGTCTTAC | qRT-PCR of 1.14.14.82 |
| c34902_g1-F | GGCAGTCAAGTAGACATGGAG | qRT-PCR of 2.3.1.170 |
| c34902_g1-R | ACCGTTTATCCGAGGAGAGTA | qRT-PCR of 2.3.1.170 |
| c41436_g1-F | CCCTCTATGATCAGCACAAGTC | qRT-PCR of 1.14.14.91 |
| c41436_g1-R | TAGGCACCATTGCCCTTTAG | qRT-PCR of 1.14.14.91 |
| c47509_g1-F | AGGCTACTCCCTTCTAG | qRT-PCR of 2.1.1.104 |
| c47509_g1-R | CTCCTGCTTCCTTGAT | qRT-PCR of 2.1.1.104 |
| c59082_g1-F | ACACAGTGGTACTGTTGATGAG | qRT-PCR of 2.3.1.74 |
| c59082_g1-R  ACTIN-F  ACTIN-R | CGATGAGTGTGAGAAGCCATAG  AATCCCAAGGCAAACAGA CCATACCAGAATCCAG | qRT-PCR of 2.3.1.74  qRT-PCR of the reference gene  qRT-PCR of the reference gene |
